# A Mechanistic Study of Plant and Microbial Controls over R* for Nitrogen in an Annual Grassland

**DOI:** 10.1371/journal.pone.0106059

**Published:** 2014-08-29

**Authors:** Stephanie G. Yelenik, Benjamin P. Colman, Jonathan M. Levine, Janneke HilleRisLambers

**Affiliations:** Department of Ecology, Evolution and Marine Biology, University of California Santa Barbara, Santa Barbara, California, United States of America; University of Tartu, Estonia

## Abstract

Differences in species' abilities to capture resources can drive competitive hierarchies, successional dynamics, community diversity, and invasions. To investigate mechanisms of resource competition within a nitrogen (N) limited California grassland community, we established a manipulative experiment using an R* framework. R* theory holds that better competitors within a N limited community should better depress available N in monoculture plots and obtain higher abundance in mixture plots. We asked whether (1) plant uptake or (2) plant species influences on microbial dynamics were the primary drivers of available soil N levels in this system where N structures plant communities. To disentangle the relative roles of plant uptake and microbially-mediated processes in resource competition, we quantified soil N dynamics as well as N pools in plant and microbial biomass in monoculture plots of 11 native or exotic annual grassland plants over one growing season. We found a negative correlation between plant N content and soil dissolved inorganic nitrogen (DIN, our measure of R*), suggesting that plant uptake drives R*. In contrast, we found no relationship between microbial biomass N or potential net N mineralization and DIN. We conclude that while plant-microbial interactions may have altered the overall quantity of N that plants take up, the relationship between species' abundance and available N in monoculture was largely driven by plant N uptake in this first year of growth.

## Introduction

Plant communities are known to be shaped by competition for resources such as light, space, nutrients, and water [1]–[3]. Differences in species' abilities to capture or alter these resources across space and time can drive competitive hierarchies, successional dynamics, community diversity, and invasions [4]–[6]. One of the best known attempts to describe these complex interactions with a simple set of rules is R* resource competition theory [5], [7], [8]. In this theory, Tilman [5] posits that plant species differ in their ability to deplete a limiting resource, and the species that maintains a positive growth rate at the lowest level of this resource will emerge as the competitive dominant. Following from Tilman [5], R* is the equilibrium concentration of resources necessary for the consumer (plant) species to maintain a stable population. Although seemingly simplistic, this theory has successfully predicted the outcome of plant competition in various ecosystems, including California grasslands [9]–[11], alpine meadows [12], and Midwestern prairies [13], [14].

Part of R* theory's success stems from providing clear predictions and testable hypotheses for ecologists [8], [15]. For example, it is relatively simple to quantify differences in resource depletion among species by planting monocultures and measuring resource concentrations after biomass stabilizes (R*). Determining whether R* values predict competitive hierarchies and structure communities can be accomplished by comparing these monoculture R* values with the relative abundance of those species in mixtures (i.e. after competition). A negative relationship between species' R* and their relative biomass in mixture supports R* theory, since the species depleting resources to the lowest levels are the competitive dominants [5], [13].

An open question is, what drives species-specific R* values in the first place? Differences in species R* levels for a soil nutrient may be due to differences in plant uptake, differences in how plants affect microbial nutrient cycling and sequestration, or both. However, the relative contribution of plants and microbes to the observed resource depletion remains largely untested. R* theory in terrestrial ecosystems has largely been tested in perennial communities or perennial communities subjected to annual invaders [9], [10], [12]–[14]. In such systems it is difficult to disentangle over multi-year timescales the relative contributions to R* of plant uptake, microbial nutrient cycling, and the indirect effects of plant traits such as litter quality and other plant-soil feedbacks [16].

Plants are often assumed to be the major drivers of R*. For example, in systems limited by nitrogen (N), depletion has largely been assumed to be due to plant N uptake and sequestration in plant tissues coupled with litter-mediated feedbacks[11], [14], [17]. Plant species can also alter microbial community composition, size, and activity, and thus alter available soil N concentrations through differences in plant traits such as quality of root exudates or root and leaf litter [18]–[21]. These effects of plant species on microbial N cycling rates can occur over short time scales [22], [23]. Carbon-rich root exudates from grasses, for example, have been shown to increase microbial respiration and N turnover in rhizosphere soils within 24 hours, resulting in higher N uptake by grasses [24].

However, microbes are also strong competitors for N in soils [25]–[28], given their high surface area [29]. Microbes are integral in the cycling of N in soils by breaking down large organic N molecules into smaller compounds (e.g., breaking proteins into amino acids and ammonium), and converting ammonium into nitrate [30]. While microbial biomass is typically not large, it turns over rapidly. As microbial biomass turns over, N from microbial “necromass” can become less available to plants and microbes as it is incorporated into soil organic matter and is protected from decomposition by a range of chemical, physical, and biological mechanisms [31], [32].

To test the extent to which plant or microbial controls on N availability underlie the R* theory of plant competition, we established an experiment in a California grassland ecosystem. We planted annual grass and forb species in monoculture and mixture plots and followed them over one growing season. Our focus on an establishing annual system is very different than the majority of work examining R* theory, most of which has been done over multiple years in perennial systems and does not explicitly examine the extent to which plants or microbes are driving the patterns [10], [33]. Although it has been previously established that plant-microbial feedbacks mediated by litter are associated with species' R* after several years in perennial systems [33], this has not been studied mechanistically (i.e., by explicitly examining the plant and microbial N pools and fluxes). Moreover, in the first year of community establishment in an annual system, such feedbacks are unlikely to be important drivers. Thus, examining an annual system over a single growing season allows for a detailed examination of short-term impacts of plants on microbial activity, and microbial activity on plant growth and nutrient acquisition, all in the relative absence of litter mediated feedbacks. As such, this study represents a first step in describing the mechanisms that drive patterns of resource competition in R* theory.

We have previously reported that the available soil N levels in monocultures in this experiment were correlated with the competitive hierarchy among plant species [11], and thus, that R* for N (and therefore, competition for nitrogen) strongly influences community dynamics in this system. The importance of competition for N is not surprising, given that grasslands in this locality have been shown previously to be N-limited [10], [34]. Here we examine the degree to which plant and microbial controls drive N levels in soils, and directly or indirectly influence plant-plant competitive dynamics.

**Figure 1:**
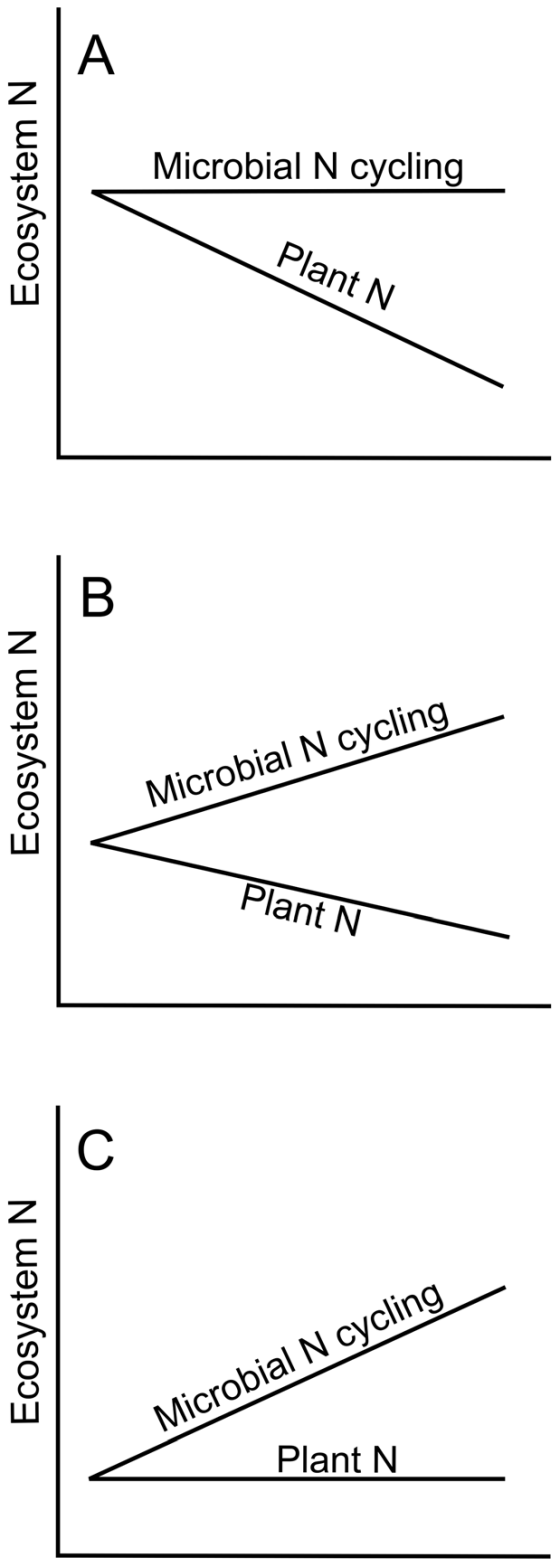
Conceptual models of plant and microbial controls of soil DIN. Our measure of R*, soil DIN, could be affected by **A** plant uptake, **B** plant uptake and microbial N cycling, or **C** microbial N cycling. See Introduction for details.

A range of different relationships could emerge between plants, microbes, and R*. If differences in R* are driven primarily by plant N uptake, we would expect to see a strong negative relationship between plant N uptake and dissolved inorganic N (DIN), our metric of R*. Furthermore, we would expect no relationship between microbial N cycling and DIN (Figure 1A). However, if differences in R* are driven primarily by changes in microbial N cycling rates (*e.g.*, net N mineralization), we would expect that microbial N cycling would be positively correlated to DIN, while plant N uptake would have no relationship with DIN (Figure 1C). If differences are driven by both differences in plant uptake and in microbial N cycling, we would expect a pattern somewhere in between the plant dominated and microbe dominated patterns with a slight positive correlation between microbial N cycling and DIN, and a slight negative relationship between plant N uptake and DIN (Figure 1B).

While we expected that both direct plant uptake of DIN and microbial N cycling would contribute to R*, we were interested in where on the continuum of plant to microbial control this annual ecosystem would fall (Figure 1A–C). Across the different monoculture and bare plots, we measured: 1) pool sizes of total N in plants; 2) DIN at the end of the growing season (our measure of R*) and over time; 3) microbial pools and process rates (microbial N, microbial biomass, net N mineralization, gross NO_3_^−^ production, and nitrification potential). To tease out drivers we then examined relationships in bare and monoculture plots between DIN and: plant biomass; microbial biomass and biomass N; and microbial process rates.

## Materials and Methods

### Site and Experimental Design

Experimental plots were established in a pasture/grassland with permission from the owner, the Midland School, in Los Olivos, California. The climate is Mediterranean, with hot, dry summers and cool, wet winters and an average annual rainfall of 550 mm. The land had not been plowed or tilled since 1940, but these soils were not undisturbed; gopher activity causes extensive physical turnover of surface soils in California grasslands, and has been estimated to give complete turnover of surface soils every 3–5 years [35]. Plots have also been grazed by cattle annually, presumably also causing soil disturbance. The vegetation is a mixture of annual and perennial herbaceous forbs and grasses growing with occasional oak trees. Soils are typic Argixerolls, with gravelly fine sandy loam texture.

Study species included three native annual forbs (*Amsinkia menziesii, Calandrinia ciliata, Clarkia purpurea*), three native annual grasses (*Muhlenbergia microsperma, Vulpia microstachys,* and *Vulpia octoflora*), and six exotic annual grasses (*Avena barbata, Bromus hordeaceous, Hordeum murinum, Lamarkia aurea, Polypogon monspielensis* and *Vulpia myorus*). All species were present in nearby grasslands (Stan Harpole, personal communication) and thus our study plants reflect a realistic representation of current California annual species. All data were collected in winter, spring, and summer of 2006.

Five ‘blocks’ were established within the grassland and were separated by 50 to 500 m. Vegetation was cleared by spraying with Roundup in fall 2005 and roto-tilling with a tractor two weeks after plants had died. Within each block, fourteen 0.64 m^2^ plots were established separated by 1 m buffers, and plots were randomly assigned treatments (species monocultures, mixtures, or bare plots). There was one full replicate of the experimental design within each block, consisting of one bare, one mixture (with all 12 species), and 12 monoculture plots, giving a total of five replicate plots for each plot type between the five blocks. Each block was fenced to exclude cattle grazing (see [11] for further details). Data from mixture plots was used to establish the R* relationship in HilleRisLambers et al. [11], by relating relative biomass in mixture to DIN in monoculture plots. Because we were trying to determine what drives DIN concentrations in the monoculture plots used to test R* theory, we focused on monoculture plots only for this study.

To establish plots, we collected seeds locally (*A. barbata, B. hordeaceous, H. murinum, L. aurea*, and *V. myorus*) or used seed from local seed companies (*A. menziesii, C. ciliata, C. purpurea, M. microsperma, P. monspielensis, V. microstachys*, and *V. octoflora*). We added a total of 15 grams of seed/m^2^ to each plot, with that weight being divided equally among the species in mixture plots. Seeds were added at the beginning of the growing season (late November 2005) and plots were watered with a volume equivalent to 3 inches of rainfall to encourage germination and establishment. We weeded plots twice to remove nontarget species, once soon after germination and once midway through the growing season. Seeding amounts in monoculture and mixture plots were sufficiently high to generate plots with little bare ground visible for all but two species (JHRL, personal observation), including *M. microsperma*, which failed to germinate. *M. microsperma* thus served as a type of bare plot, and may be considered as a more appropriate control because it had an initial N input from seed, similar to other planted plots. We refer to it as bare (seeded) in the rest of the manuscript and figures.

### Soil N Pools and Dynamics

We collected all soils immediately after plant biomass harvest. We sampled soil by extracting two 5 cm diameter, 10 cm depth soil cores from each plot. From each of the five replicate plots for each species, duplicate cores were sieved (2 mm mesh size) prior to soil and microbial analyses (see *Microbial N Pools and Process rates*, below). Dissolved inorganic N (DIN hereafter) was quantified by extracting ammonium and nitrate (NH_4_^+^ and NO_3_^−^ ) with 2M KCl which were then analyzed on a Lachat flow injection autoanalyzer (Lachat Instruments; Loveland, USA), while soil moisture was determined gravimetrically after six days drying at 60°C. DIN was our measure of R* [11]. Total soil N pool (which includes DIN, as well as organic N) was measured on dried soils after they were ground in a ball mill (Wig-L-Bug amalgamator, Crescent Dental, Lyons IL), after which Carbon (C) and N content were measured on a Carlo Erba NA 1500 CHN analyzer (Fisons Instruments, Beverly, MA).

Soil bulk density was measured in June by collecting five soil cores randomly placed within plots in each block (4 cm diameter, 10 cm deep). These were returned to the lab, dried for 48 h at 60°C, rocks were removed, and remaining soil weighed. Block-specific bulk densities were used to convert all soil measures to g m^−2^ unit values to enable comparison of N budgets between soils, plants, and microbes. Outcomes of our statistical analyses on soil N pools did not differ given different N measurement units (i.e., g N m^−2^ versus g N kg^−1^ soil).

To determine whether our R* measure (DIN pools in May) was representative of N dynamics over the growing season, we used resin stakes (PRS Probes; Western Ag Innovations, Saskatoon, Canada) to track inorganic N levels in soils throughout the experiment [36]. Plastic stakes with ion exchange resin surfaces were placed in 0–5 cm soils in mid-month February 2006, and switched each month thereafter until mid-month June 2006. Thus the May sample date represents resin-available N sampled between mid-April and mid-May. Excess soil was washed off of probes with DI water. Probes were then extracted in 0.5 M HCl, and extracts were analyzed for NH_4_^+^ and NO_3_^−^ as described above. Regenerated stakes were placed into the same slots in the soil as previous stakes.

### Plant N Pools

Plant biomass was harvested in mid-May for all species; this timing was chosen based on close monitoring of the diverse phenologies of the species in the experiment. At this time, all species had reached peak biomass and set seed, but had not senesced. Aboveground plant biomass was sub-sampled in each plot by clipping all vegetation within one 10×50 cm quadrat. Roots were collected from duplicate soil cores collected within the clip plots by sieving soils (see above in *Soil N Pools and Dynamics*) in each of the five replicate plots for each vegetation type. All plant material was dried at 60°C for six days and weighed. Plant material was ground with a Wiley mill (Thomas Scientific, Philadelphia, PA) followed by a ball mill. Plant samples were then analyzed for C and N on the CHN analyzer. Total plant N (g N/m^2^) in monoculture plots was then quantified as (aboveground biomass/m^2^ × shoot % N/100) + (belowground biomass/m^2^ × root % N/100).

### Microbial N Pools and Process Rates

Prior to examining microbial biomass and N cycling, sieved soils were adjusted to 35% water holding capacity (WHC) to eliminate the confounding effects of variable water content on process rates. Soils were allowed to equilibrate for 7 days to allow the effects of sieving and drying/rewetting to pass, after which soils were weighed out for all analyses. Lab replicates were not included given that soils were sieved, there were five field replicates per treatment, and past work using these same techniques in soils from this area showed tight agreement among lab replicates [37].

To examine how microbial biomass N relates to R*, we used a chloroform slurry extraction to examine the flush of N released from microbial cell lysis and extraction [38]. Briefly, 25 mL of 0.5 M K_2_SO_4_ was added to each glass tube containing a 4 g soil sample. To this, 0.5 mL of EtOH-free chloroform was added. Tubes were sealed with PTFE lined caps, and shaken for 4 h at 150 rev min^−1^ on an orbital shaker. Tubes were allowed to settle for 10 minutes allowing the bulk of the chloroform and soil slurry to separate. The top 10 mL of extract was filtered through a Pall A/E glass fiber filter (Pall Corporation, Port Washington, NY, USA), then bubbled with air for 20–30 minutes to remove any residual chloroform. Chloroform slurries were compared to slurries extracted without chloroform.

To test the influence of microbial abundance on R* we used substrate induced respiration (SIR), an index of microbial biomass [39]. Briefly, 10 mL of a 12 g L^−1^ Difco Yeast Extract (Becton, Dickinson and Company, Franklin Lakes, NJ, USA) was added to 4 g soil. Soils were capped and shaken for four hours with 1 mL headspace CO_2_ subsamples withdrawn at 20 minutes, two hours, and four hours. Gas samples were analyzed on a LI-6262 infrared gas analyzer (LI-COR, Lincoln, NE, USA). The SIR biomass was then calculated by applying a linear fit to the change in CO_2_ over time (average r^2^ = 0.99).

Because microbial biomass turnover rates are faster than those of annual plants, microbes may have driven inorganic N availability in soils via process rates that are not reflected in microbial biomass pools. Thus, we also quantified potential net N mineralization, gross NO_3_^−^ production, and nitrification potential. Net N mineralization is the net accumulation of extractable NH_4_^+^ and NO_3_^−^ in soil, and represents the balance between microbial production and consumption. Gross nitrification separates microbial production and consumption of NO_3_^−^, and offers insight into nitrifier abundance and ambient substrate (NH_4_^+^) availability. In contrast to gross rates, nitrification potential assays add NH_4_^+^ in excess and thus rates are driven by—and serve as an index of—the abundance and potential activity of ammonia oxidizers. Net accumulation of inorganic N is associated with conditions where heterotrophic microbes are not limited by N availability, while high gross rates of nitrification and high nitrification potential are associated with high availability of NH_4_^+^ and NO_2_^−^ to autotrophic nitrifiers.

For net N mineralization, two sets of 4 g samples were used, with one set extracted on the first day of incubation for one hour with 25 ml of 0.5 M K_2_SO_4_, while the other set was incubated for 60 days at 20°C prior to extraction [37]. Net N mineralization was then calculated as the difference in extractable NH_4_^+^ and NO_3_^−^ in soils between the initial and final time points. These rates can be seen as potential rates because they were held at optimal moisture and temperature for microbial activity. Our method has the advantage of controlling for microclimate variability that may affect mineralization rates, but is not expected to perfectly match field rates.

To quantify gross nitrification, we took duplicate 4 g samples of soil at 35% WHC and added 0.25 ml of 25.5 mg N/L (98% enriched ^15^N-NO_3_^−^) stirring with the pipette tip after addition. Samples were then extracted for one hour with 25 ml of 0.5 M K_2_SO_4_, with one sample extracted at 15 min, and the other sample at 24 h. After NO_3_^−^ was measured, extracts were prepared for isotope ratio mass spectrometry. First, NH_4_^+^ was converted to NH_3_ with MgO and driven out of solution. Then, NO_3_^−^ was converted to NH_3_ by Devarda's Alloy in the presence of MgO, and the NH_3_ was then captured on acidified filter disks in teflon packets using the methods of Sørensen and Jensen [40]. Filter packs were removed and placed in a desiccator with DrieRite and concentrated H_2_SO_4_ to dry filters and trap any free NH_3_. Filters were folded into tin capsules, and ^15^N enrichment was measured at UCSB's Marine Science Institute Analytical Lab using a Thermo-Finnigan MAT Delta+ Advantage (Thermo Fisher GmBH, Dreieich, Germany). Gross nitrification rates were calculated following Hart et al. [41]. We aimed to measure gross N mineralization in addition to gross nitrification, however NH_4_^+^ pools in soils were too low, and consumption too high, such that it precluded quantifying gross N mineralization.

Nitrification potential was measured by adding 35 ml of a stock solution containing 8 mL 0.2 M K_2_HPO_4_, 1 ml 0.2 M KH_2_PO_4_, 5 ml 0.2 M (NH_4_)_2_SO_4_, and 20 ml of 1 M NaClO_3_ (added to block the activity of nitrite oxidizers; [42]). The rate of appearance of nitrite was measured at three time points over 4 h using a modified Griess-Illosvay reagent [43], with 1 ml of color reagent added to 4 ml of sample.

### Statistical Analyses

We asked what the patterns in DIN were over the course of the growing season under our different monoculture types (i.e. plant community composition, time) by fitting the resin N data with five linear generalized mixed effects models (with block as random effect in all), including: a null model; a model with only species identity as explanatory variable; a model with only time as the explanatory variable; a model with both time and species identity as explanatory variables; and a model with the main effects of time and species identity as well as their interaction. Specifying block as a random effect with time as an explanatory variable is analogous to a repeated measures ANOVA. The log of resin N values was used as our response variable to ensure normality. We used AIC's to determine the best-fitting of the five models, and the appropriate likelihood ratio tests to determine the significance of explanatory variables retained in the best fitting model. We also used resin-N data to test whether our one time measure of soil DIN represented soil dynamics across the season, by correlating species-specific cumulative resin N values for each species (over the entire growing season) to species-specific DIN values. We used Kendall's tau rather than Pearson's r for this correlation because both DIN values were non-normally distributed.

We also asked whether DIN values were greater in plots without any vegetation, as we would expect if plants influence available soil N. We did this using a generalized mixed effects model with DIN (log transformed) as the response variable and the categorical explanatory variable ‘vegetated’ vs. ‘bare’.

We then asked how species-specific DIN related to N pools and fluxes. First we used 1- way ANOVAs with species as a fixed effect and block as a random effect to ask whether plant and microbial parameters were different between species (Table 2). We then quantified the correlation between DIN and either: total plant N; Microbial N; Microbial biomass (substrate induced respiration, SIR); Net N mineralization; Nitrification potential; and Gross nitrification (we use the term “microbial biomass/process rates” to encompass these measurements of biomass or process rates hereafter). Kendall's tau was used to assess the relationship between these variables since many were non-normally distributed. If plant uptake was driving N dynamics, we expected to find a negative relationship between DIN and plant N, and no clear relationship between DIN and other N pools and fluxes (Figure 1A). By contrast, if microbial processes were driving R*, then we would expect to find positive correlations between microbial biomass/process rates and DIN (our proxy for R*), but no clear relationship between plant biomass N and DIN (Figure 1C). If a combination of microbial cycling and plant uptake were driving DIN, we would expect a modest positive correlation between microbial biomass/process rates and DIN, and a modest negative correlation between plant biomass N and DIN (Figure 1B).

**Table 2:** Effect of plant species on soil N pools and process rates.

Response Variable	F	df	P
Total Soil N (g N m^−2^)	1.41	11,49	0.20
DIN (g N m^−2^)*	2.63	11, 49	0.01
Microbial Biomass N (g N m^−2^)	0.77	11, 49	0.67
Microbial (SIR) Biomass (ug CO_2_ g^−1^ soil hr^−1^)	1.30	11, 49	0.25
Potential Net N Mineralization (µg N m^−2^ d^−1^)	1.90	11, 49	0.06
Nitrification Potential (µg N g^−1^ soil h^−1^)*	1.90	11, 49	0.06
Gross Nitrification (µg N g^−1^ soil d^−1^)*	2.56	11,40	0.01
Plant N (g N m^−2^)	3.14	10,43	<0.01

(ANOVA with species as fixed effect and block as a random effect).

*log transformed for normality.

While examining the longer term effects of litter mediated plant-soil feedbacks is beyond the scope of this study, we used linear regression to examine the relationship between plant C:N and plant biomass to provide some insight into potential relationships. A positive correlation between biomass and litter C:N would reinforce the short-term R* patterns, with plants that reach high biomass and draw N levels to the lowest levels further depleting soil N with C rich and N poor litter. A negative correlation could suggest that litter-feedbacks might drive the system in the opposite direction, with plants that achieve lower biomass and have a higher R* potentially slowing decomposition over longer time scales with high C:N litter.

Finally, we asked how plant productivity (as measured by total plant biomass per m^2^) related to total available N pools (summed over soils, microbes and plant biomass). We used Kendall's tau for these correlations. All analyses were performed using R version 2.13.1 [44].

## Results

### N Pools and Dynamics

In examining N pools in this annual grassland, total soil N accounted for 154±4 g N/m^2^. Microbial biomass N and DIN pools (NO_3_^−^+NH_4_^+^) comprised a small fraction of soil N pools, with each accounting for about 0.3% of the total soil N pool (Table 1). In contrast to microbes, plants were a much larger N pool, about ten-fold higher than microbial biomass N (Table 1).

**Table 1:** Soil (0–10 cm), microbial, and plant N pools.

Nitrogen Pool	g N/m^2^	% of Total Soil N
Total Soil N	154±4	
Soil NH_4_^+^+NO_3_^−^	0.47±0.05	0.3
Microbial biomass N	0.47±0.03	0.3
Plant N (above + below)	4.43±0.33	3.0

*Values represent averages across all treatments ± standard error.

**Figure 2:**
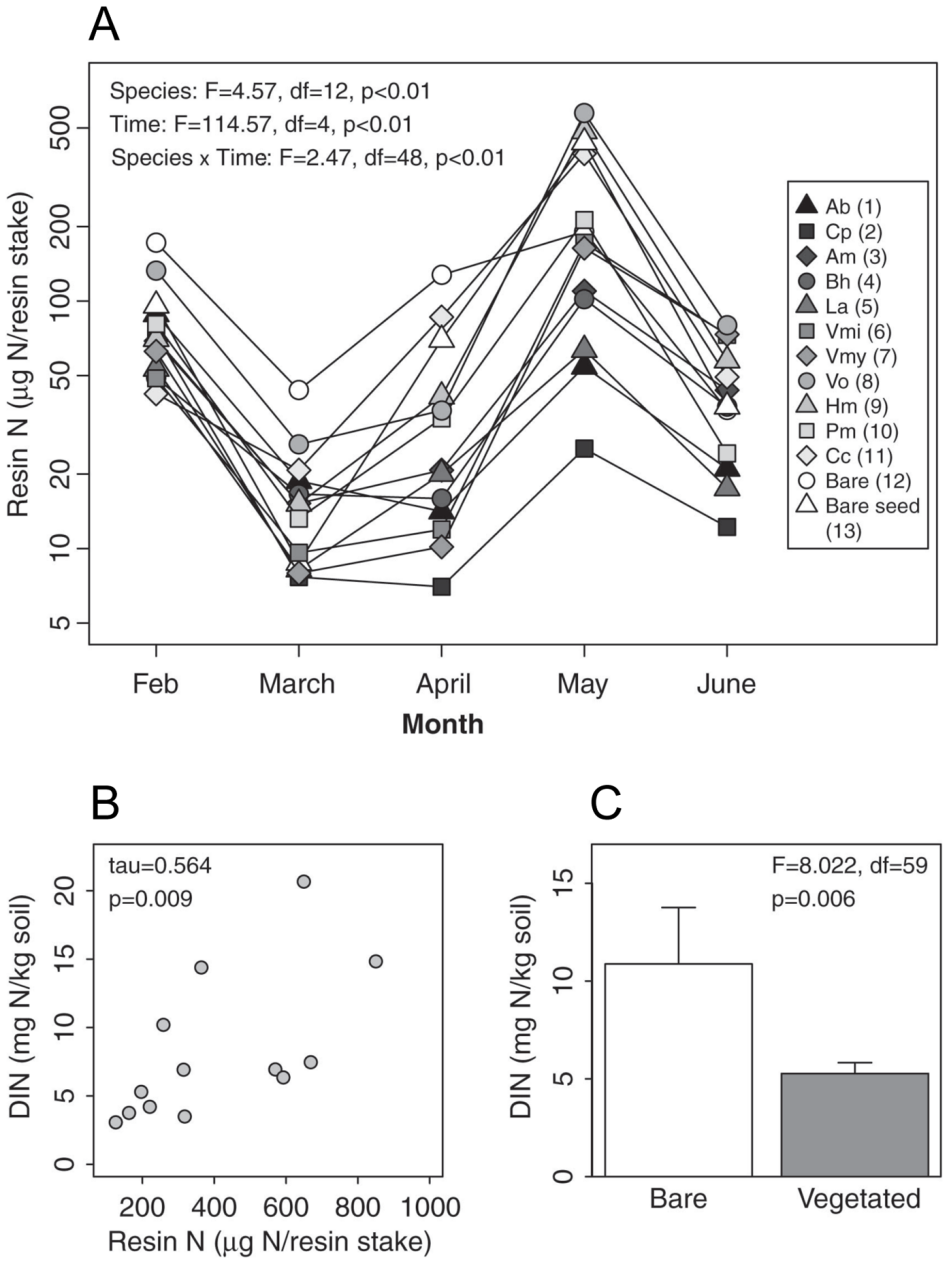
Resin available N in soil during growing season. **A** Point measures over time in monoculture and bare plots. Species are listed in order of monoculture aboveground biomass and this rank is noted in parenthses (1 = greatest biomass, 12 = lowest biomass) and denoted with increasing grayscale values for symbols indicating greater plant biomass. **B** Relationship between cumulative resin-available N (summed over time) and final DIN levels (R*) in monoculture and bare plots. **C** Final DIN in bare and planted plots. Data were log transformed to account for non-normality prior to analyses, and bars show backtransformed coefficients (±1SE) reflecting bare and planted DIN means. Species abbreviations: *Amsinkia menziesii* (Am), *Avena barbata* (Ab), *Bromus hordeaceous* (Bh), *Calandrinia ciliate* (Cc), *Clarkia purpurea* (Cp), *Hordeum murinum* (Hm), *Lamarkia aurea* (La), *Polypogon monspielensis* (Pm), *Vulpia microstachys* (Vmi), *Vulpia myorus* (Vmy) and *Vulpia octoflora* (Vo). Bare seeded plots received seed addition, but did not germinate (see methods).

Our indicator of growing season N dynamics, resin N, varied by species over time for all monoculture plots. A model including species identity, time, and their interaction gave the best fit (Figure 2A), as indicated by AIC values (data not shown). Resin available N increased in May in all plots, with a pronounced increase in both seeded bare plots as well as plots with low plant biomass (*Vulpia octoflora*, Vo; *Hordeum murinum*, Hm; and *Calandrinia ciliata*, Cc). In contrast, this response was tempered in monoculture plots with high plant biomass (e.g., *Clarkia purpurea*, Cp).

The five-month sum of resin-available N was significantly correlated with DIN pools measured in May (Figure 2B). This shows that our R* measure (May DIN pools) was correlated with the soil N dynamics through the growing season. The temporal trends in the resin N data showed that plots with high plant biomass maintained lower DIN. Consistent with these data, we found that vegetated plots had lower DIN (R*) values than bare plots (Figure 2C).

### Plant N Uptake

**Figure 3:**
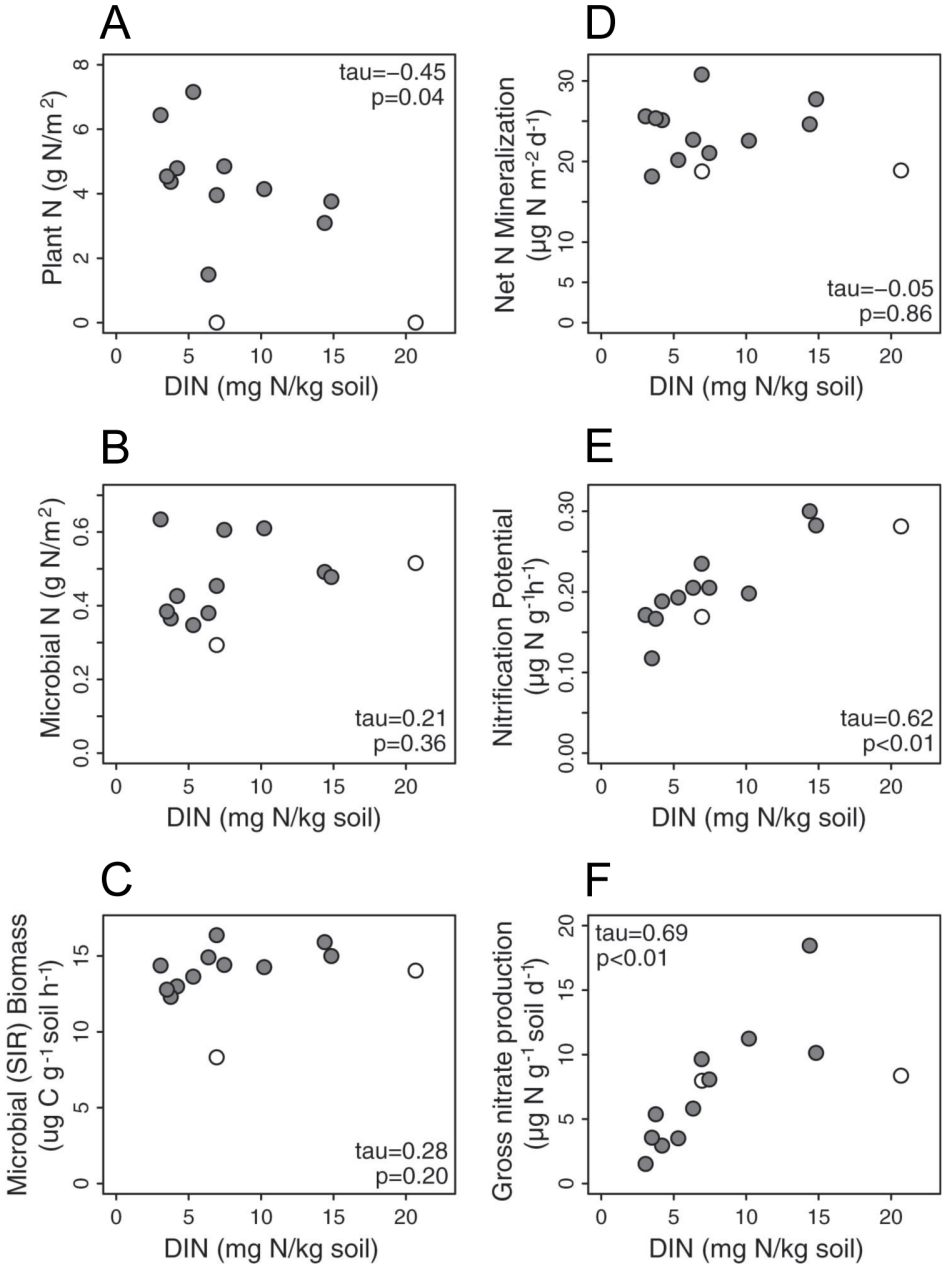
Comparison of soil DIN and plant and microbial metrics. **A** total plant N, **B** Microbial N, **C** Microbial biomass measured by substrate induced respiration (SIR), **D** Potential net N mineralization, **E** Nitrification potential, and **F** Gross nitrification for monocultures and bare plots. White dots represent bare and bare seeded plots, grey dots are vegetated plots.

**Figure 4:**
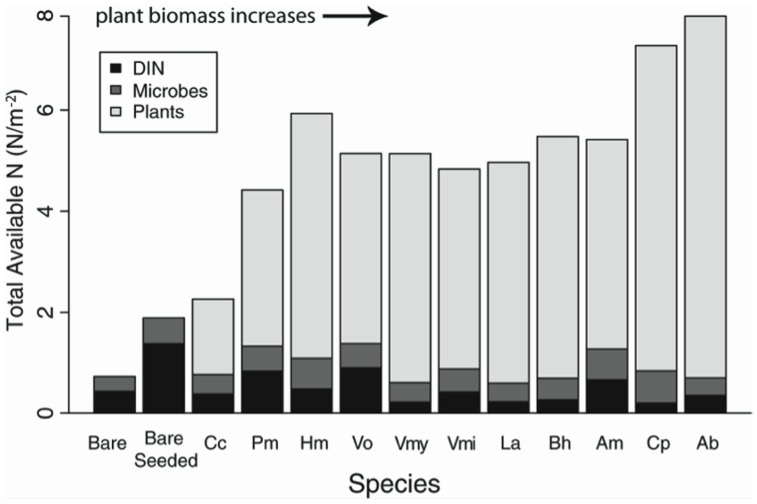
Sum of plant N, microbial N, and final soil DIN to find a total available N in each monoculture plot. Species are listed left to right in order from lowest to highest ranking of aboveground plant biomass in monoculture plots. Kendall's tau between total available N per m^2^ (sum of microbial, plant and soil) and above ground plant biomass is 0.68, 2-sided p = 0.001. Species abbreviations are as in Figure 1.

Monoculture plant biomass N was significantly and negatively correlated with soil DIN (Figure 3A), and gave a similar trend with resin-available N. Monoculture plots with high plant N (e.g., *Clarkia purpurea*) consistently exhibited lower levels of resin-available N than bare plots (Figure 2A). When comparing N pools of plants and microbes within monoculture plots, even for those plants that took up very little N (*e.g., C. ciliata*), plant biomass was still a larger N pool than microbial biomass. This difference became greater with increasing aboveground plant biomass (Figure 4).

We found that plant species differed in their uptake of N into plant biomass. Differences in plant biomass N were related to differences in total plant biomass yielding a strong positive correlation between the two (r^2^ = 0.81, p<0.001, n = 54), whereas plant % N showed a slight negative correlation with N plant biomass N (r^2^ = 0.10, p = 0.02, n = 54). Plant species that obtained higher biomass in monoculture plots also had higher C:N ratios (C:N aboveground biomass versus biomass in monoculture: r^2^ = 0.67, p = 0.007, data not shown).

### Microbial N Uptake and Process Rates

Microbial biomass N and SIR biomass did not differ in soils across plant monoculture plots (Table 2), and had no relationship with DIN (Figs. 3B, C). Similarly, there was no relationship between DIN and our indicator of microbial N cycling rates, net N mineralization (Figure 3D). Our two metrics of the conversion of NH_4_^+^ to NO_3_^−^, nitrification potential and gross nitrate production, did show significant positive correlations with DIN (Figs. 3E, F).

## Discussion

While a number of studies have shown that a plant species' depletion of soil available N can be predictive of its competitive ability in mixture, the extent to which plants or microbes drive the N depletion has remained largely unexplored. Set in the context of an N-limited annual grassland ecosystem [10], [34], our study suggests that plant N uptake was more important than species-specific effects on microbial processes in driving plant species' differences in their R* (Figure 1A), and thus uptake determined their competitive dominance in the system [11]. Three lines of evidence support this interpretation: 1) plant N and soil DIN were negatively correlated across the monocultures of different plant species (Figure 3A); 2) microbial N was not correlated with soil DIN (Figure 3B); and 3) net N mineralization was not correlated with soil DIN (Figure 3D).

### Evidence that Plants Wear the Pants

The negative correlation between plant N and soil DIN (Figure 3A) suggests that plant uptake was largely responsible for the observed species level differences in R* at the end of the growing season. Additionally, throughout the growing season most of the monocultures—especially those species that achieved high biomass—maintained lower levels of resin-available N than bare plots, where N levels were primarily a function of microbial processes (Figure 2C).

We found that neither microbial biomass N nor net N mineralization was correlated with soil DIN at the end of the growing season (Figure 3B–D) This suggests that microbes were not driving differences in soil DIN via direct uptake and immobilization of available N into microbial biomass, and that microbial processing of organic N was not driving differences in DIN levels across plant species. It has been suggested in the literature that, while microbes do not drive available N via direct uptake, they do regulate N via processing and plants take up the “leftovers” [27]. If this were the case in the present study net N mineralization would be expected to positively correlate with plant N uptake [27], which is a relationship we did not find in our system (net N mineralization versus plant N uptake: r^2^ = 0.05, p = 0.47).

In fact, nitrification potential and gross nitrification were the only measured microbial parameters that were correlated with DIN (Figs. 3E, F). Nitrification is a dissimilatory process and thus only alters N form, not DIN pool size. Since nitrifier abundance (nitrification potential) and process rates (gross nitrification) correlated to substrate pools (DIN, Figs. 3E, F), we hypothesize that they were limited in their activity by the size of their substrate pools of NH_4_^+^ and NO_2_^−^ [45], [46]. This is consistent with the current understanding of these chemoautotrophic organisms, which are generally considered poorer competitors for soil N than heterotrophic microbes or plant roots [46].

In contrast, the heterotrophic microbes which drive N mineralization and immobilization were likely not limited by N availability. Rather, they functioned similarly across monoculture plots regardless of N uptake by plants and DIN concentrations (Figs. 3B–D). This is consistent with low soil C:N values, which averaged 7.3 (±0.1), indicating that heterotrophic microbes were more limited by C than N [47]. C-limitation in turn may explain why heterotrophic microbes did not seem to drive final DIN levels.

### Evidence of plant-microbial interactions

Our data suggest that plant N uptake drove DIN pool sizes in this annual system, and thus drove R*. This is consistent with ^15^N tracer experiments which postulate that plants immobilize a greater amount of N into plant biomass over an entire growing season, and thus drive the longer-term patterns of N fate in ecosystems [25], [26], [29], [48]. However, these same studies make it clear that microbes can outcompete plants for available soil N in the short term, with some of the best evidence for this coming from California grasslands [25], [26].

Our goal was to test the mechanisms underlying R* by determining the role of plant uptake as compared to changes in microbial biomass and microbially mediated N cycling rates. Understanding such mechanisms is particularly important in such ecosystems where competition outcomes among annuals in the first year of community assembly are likely to be important for long-term community trajectories. By constraining our study to one year in an annual system, we explicitly removed the confounding effect of plant-microbe feedbacks as mediated by litter, which are more likely to develop over several years [20]. Short-term competition stands to be particularly important in these California grasslands, where disturbance plays a large role in exotic annual grass invasion [10], [11] and the dominant competitors are annual.

**Figure 5:**
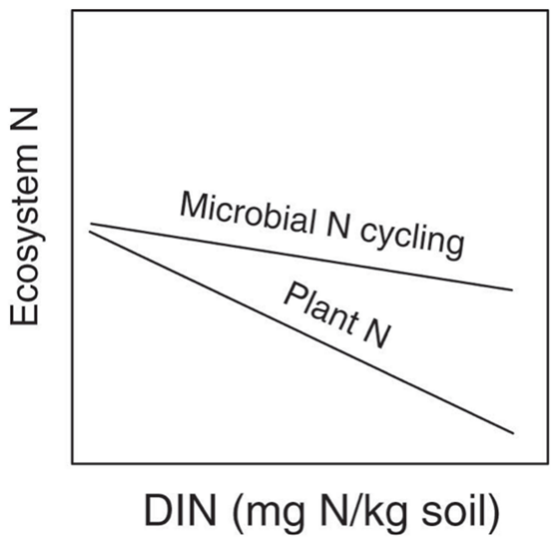
Revised conceptual model plant and microbial contributions to driving DIN (our indicator of R*) in this annual system. In contrast to models shown in Figure 1, in this model, DIN decreases as plant N increases due to plant uptake (Figure 3A), similar to earlier predictions (Figure 1A,B). In addition, greater plant biomass N may have been obtained through stimulation of microbial N cycling (Figure 4), in contrast to earlier predictions (Figure 1B,C).

While our data suggest that plant N uptake drove soil DIN pools, another story emerges when the focus is shifted from looking at comparisons of individual pool sizes and fluxes (Figure 3) to looking at the total N in plants, microbes, and DIN for each set of monoculture plots (Figure 4). Examining the sum of N in plants, microbes, and DIN reveals that there were larger pools of biological and/or bioavailable N in plots with higher plant biomass (Figure 4). This suggests that microbes released more plant available N over the course of the growing season in those plots with higher plant biomass and lower R*, even though our measure of microbial N turnover, net N mineralization, did not differ between plots. Comparing planted vs. bare plots, there was more N in these pools (plant N, microbial N, and DIN) in planted (5.4±0.3 g N/m^2^) than bare plots (1.3±0.7 g N/m^2^; F_1,62_ = 26.7, p<0.001) and total N in these pools increased with increasing plant biomass (Figure 4) and decreasing R*. Thus, while plant N uptake was working in a manner consistent with our conceptual models, microbial N cycling may have actually been responding in the opposite direction of our predictions. In other words, microbial N cycling in the field may have been negatively, rather than uncorrelated or positively correlated with DIN (Figure 5); in plots with lower R* and higher plant N uptake, microbes may have generated more plant available N than in plots with higher R* and lower plant N uptake.

It is possible that this pattern—where the sum of plant N, microbial N, and DIN was higher in plots with lower R*—could be explained by large losses of available N from bare plots due to denitrification or leaching. Several lines of evidence suggest that these explanations are unlikely. Denitrification depends on labile C, which would have led to higher potential for N losses due to denitrification in planted plots with roots exuding labile C as opposed to those that were bare or had sparse cover. Thus denitrification would be expected to yield more loss of N with increasing biomass, which would lead to a pattern opposite to that observed in the total N pools (Figure 4). While leaching losses would have been higher in plots with lower biomass due to decreased evapotranspiration, we suggest it is unlikely to have been the main driver of the observed patterns. Over 94% of the annual rainfall had fallen by mid-April, prior to peak plant growth and strong differences in monoculture soil resin available N (Figure 2A). The two remaining rainfall events were small enough that they would not have caused significant leaching.

While we cannot rule out denitrification and leaching, we hypothesize that plant ‘priming’ of microbes led to differences in microbial N cycling in the field, which in turn altered plant uptake of DIN, and thus the total distribution of N in different ecosystem compartments. Priming is the stimulation of microbial activity via labile C-rich root exudates. The low C:N ratio of these soils makes it likely that microorganisms were limited more by C than by N [47]. Plant roots releasing labile C into rhizosphere soil may have led to localized increases in microbial activity [49] leading to higher N availability in rhizosphere soil [21], [50]. Because we did not separate rhizosphere from bulk soil, localized increases in microbial activity, such as net N mineralization, were less likely to be observed [50], [51]. Priming has been shown to increase plant N uptake in various ecosystems [24], [50]–[52] including California grasslands, where it was shown to increase gross N mineralization rates by ten-fold [51]. Thus plant-microbe interactions may have increased N cycling in this current experiment in plots with lower R* (Figure 5), but our measures of N cycling were not sensitive to those differences given several factors, including: the localized nature of this interaction; the removal of roots and fresh C inputs which would have been the drivers of this interaction; and the short preincubation following sieving and adjustment of water content.

While our results were consistent with plants driving patterns in R* through uptake of N (Figure 3A), and showed no evidence of microbial processes or biomass driving R* (Figure 3A–C), when ecosystem N pools are taken as a whole (Figure 4) those plots that accumulated more N in microbial, plant, and DIN pools likely had increased cycling and availability of N to plants. This suggests that even over the course of one growing season, plant-microbial interactions (*e.g.*, priming) may be important to ecosystem N cycling and ultimately, may influence plant competition.

It remains an open question as to whether these patterns would persist over multiple growing seasons, and what kind of impact litter mediated feedbacks would have in this annual system. In our experiment, plant species that obtained higher biomass in monoculture plots also had lower quality biomass (higher C:N in aboveground biomass), suggesting that the patterns observed in this study would be reinforced over multiple years by litter mediated feedbacks. It is likely that monoculture plots of annual species with high N use efficiency and low N litter would depress N mineralization, helping drive low R* (Figure 1B,C) and the magnitude of this effect could increase with time as feedbacks become better established. Thus for annuals growing in continuous monoculture, we would expect to see similar patterns to perennial systems [12], [33], where the persistence of plants allows for strong litter feedbacks that help determine plant species' R* for nitrogen.

## References

[1] Grime JP (1977) Evidence for existence of three primary strategies in plants and its relevance to ecological and evolutionary theory. American Naturalist 111: 1169–1194.

[2] Connell JH (1983) On the prevalence and relative importance of interspecific competition: evidence from field experiments. American Naturalist 122: 661–696.

[3] Pacala SW, Tilman D (1994) Limiting similarity in mechanistic and spatial models of plant competition in heterogeneous environments. American Naturalist 143: 222–257.

[4] Connell JH, Slatyer RO (1977) Mechanisms of succession in natural communities and their role in community stability and organization. American Naturalist 111: 1119–1144.

[5] Tilman D (1982) Resource competition and community structure. Princeton, NJ: Princeton University Press. 7162524

[6] Goldberg DE (1990) Components of resource competition in plant communities. In: Grace J, Tilman D, editors.Perspectives on plant competition.San Diego: Academic Press. pp. 27–49.

[7] Tilman D (1985) The resource-ratio hypothesis of plant succession. American Naturalist 125: 827–852.

[8] Miller TE, Burns JH, Munguia P, Walters EL, Kneitel JM, et al (2005) A critical review of twenty years' use of the resource-ratio theory. American Naturalist 165: 439–448. 10.1086/428681 15791536

[9] Dukes JS (2001) Biodiversity and invasibility in grassland microcosms. Oecologia 126: 563–568. 28547241 10.1007/s004420000549

[10] Seabloom EW, Harpole WS, Reichman OJ, Tilman D (2003) Invasion, competitive dominance, and resource use by exotic and native California grassland species. Proceedings Of The National Academy Of Sciences 100: 13384–13389. 10.1073/pnas.1835728100 PMC263823 14595028

[11] HilleRisLambers J, Yelenik SG, Colman BP, Levine JM (2010) California annual grass invaders: the drivers or passengers of change? Journal Of Ecology 98: 1147–1156. 20852668 10.1111/j.1365-2745.2010.01706.x PMC2936119

[12] Suding KN, Larson JR, Thorsos E, Steltzer H, Bowman WD (2004) Species effects on resource supply rates: do they influence competitive interactions? Plant Ecology 175: 47–58.

[13] Fargione J, Tilman D (2006) Plant species traits and capacity for resource reduction predict yield and abundance under competition in nitrogen-limited grassland. Functional Ecology 20: 533–540.

[14] Harpole SW, Tilman D (2006) Non neutral patterns of species abundance in grassland communities. Ecology Letters 9: 15–23. 16958864 10.1111/j.1461-0248.2005.00836.x

[15] Fox JW (2002) Testing a simple rule for dominance in resource competition. American Naturalist 159: 305–319. 10.1086/338543 18707382

[16] Hodge A, Fitter AH (2013) Microbial mediation of plant competition and community structure. Functional Ecology 27: 865–875.

[17] Tilman D, Wedin D (1991) Plant traits and resource reduction for five grasses growing on a nitrogen gradient. Ecology 72: 685–700.

[18] Eviner VT (2004) Plant traits that influence ecosystem processes vary independently among species. Ecology 85: 2215–2229.

[19] Hawkes CV, Wren IF, Herman DJ, Firestone MK (2005) Plant invasion alters nitrogen cycling by modifying the soil nitrifying community. Ecology Letters 8: 976–985. 10.1111/j.1461-0248.2005.00802.x 34517683

[20] Yelenik SG, Levine JM (2010) Native shrub re-establishment in exotic annual grasslands: do ecosystem processes recover? Ecological Applications 20: 716–727. 20437958 10.1890/08-2365.1

[21] Brzostek ER, Greco A, Drake JE, Finzi AC (2013) Root carbon inputs to the rhizosphere stimulate extracellular enzyme activity and increase nitrogen availability in temperate forest soils. Biogeochemistry 115: 65–76.

[22] Curiel Yuste J, Baldocchi DD, Gershenson A, Goldstein A, Misson L, et al (2007) Microbial soil respiration and its dependency on carbon inputs, soil temperature and moisture. Global Change Biology 13: 2018–2035.

[23] Fornara DA, Tilman D, Hobbie SE (2009) Linkages between plant functional composition, fine root processes and potential soil N mineralization rates. Journal of Ecology 97: 48–56.

[24] Hamilton EW, Frank DA (2001) Can plants stimulate soil microbes and their own nutrient supply? Evidence from a grazing tolerant grass. Ecology 82: 2397–2402.

[25] Jackson LE, Schimel JP, Firestone MK (1989) Short-term partitioning of ammonium and nitrate between plants and microbes in an annual grassland. Soil Biology & Biochemistry 21: 409–415.

[26] Schimel JP, Jackson LE, Firestone MK (1989) Spatial and temporal effects on plant-microbial competition for inorganic nitrogen in a California annual grassland. Soil Biology & Biochemistry 21: 1059–1066.

[27] Kaye JP, Hart SC (1997) Competition for nitrogen between plants and soil microorganisms. Trends in Ecology & Evolution 12: 139–143. 21238010 10.1016/s0169-5347(97)01001-x

[28] Schmidt SK, Costello EK, Nemergut DR, Cleveland CC, Reed SC, et al (2007) Biogeochemical consequences of rapid microbial turnover and seasonal succession in soil. Ecology 88: 1379–1385. 17601130 10.1890/06-0164

[29] Kuzyakov Y, Xu X (2013) Competition between roots and microorganisms for nitrogen: mechanisms and ecological relevance. New Phytologist 198: 656–669. 23521345 10.1111/nph.12235

[30] Schimel JP, Bennett J (2004) Nitrogen mineralization: challenges of a changing paradigm. Ecology 85: 591–602.

[31] Schmidt M, Torn MS, Abiven S, Dittmar T, Guggenberger G, et al (2011) Persistence of soil organic matter as an ecosystem property. Nature 478: 49–56. 21979045 10.1038/nature10386

[32] Kleber M, Sollins P, Sutton R (2007) A conceptual model of organo-mineral interactions in soils: self-assembly of organic molecular fragments into zonal structures on mineral surfaces. Biogeochemistry 85: 9–24.

[33] Wedin DA, Tilman D (1990) Species effects on nitrogen cycling: a test with perennial grasses. Oecologia 84: 433–441. 28312957 10.1007/BF00328157

[34] Harpole WS, Goldstein L, Aicher RJ (2007) Resource limitation. In: Stromberg MR, Corbin JD, D'Antonio CM, editors.California Grasslands.Berkeley, CA: University of California Press. pp. 119–127.

[35] Harrison S, Viers J (2007) Serpentine grasslands. California grasslands: ecology and management University of California Press, Berkeley: 145–155.

[36] Johnson DW, Verburg PSJ, Arnone JA (2005) Soil extraction, ion exchange resin, and ion exchange membrane measures of soil mineral nitrogen during incubation of a tallgrass prairie soil. Soil Science Society of America Journal 69: 260–265.

[37] Colman BP, Schimel JP (2013) Drivers of microbial respiration and net N mineralization at the continental scale. Soil Biology and Biochemistry 60: 65–76.

[38] Fierer N, Schimel JP (2002) Effects of drying-rewetting frequency on soil carbon and nitrogen transformations. Soil Biology and Biochemistry 34: 777–787.

[39] Fierer N, Schimel JP, Holden PA (2003) Variations in microbial community composition through two soil depth profiles. Soil Biology & Biochemistry 35: 167–176.

[40] Sørensen P, Jensen ES (1991) Sequential diffusion of ammonium and nitrate from soil extracts to a polytetrafluoroethylene trap for ^15^N determination. Analytica Chimica Acta 252: 201–203.

[41] Hart SC, Nason GE, Myrold DD, Perry DA (1994) Dynamics of gross nitrogen transformations in an old-growth forest - the carbon connection. Ecology 75: 880–891.

[42] Belser LW, Mays EL (1980) Specific inhibition of nitrite oxidation by chlorate and its use in assessing nitrification in soils and sediments. Applied and Environmental Microbiology 39: 505. 16345525 10.1128/aem.39.3.505-510.1980 PMC291368

[43] QuickChemMethod10-107-04-1-A (1995) Nitrate/nitrite, nitrite in surface water, waste water, 0.2 to 20 mg/L. In: QuickChem Automated Ion Analyzer Methods Manual: Lachat Instruments, Milwaukee, U.S.A.

[44] R Development Core Team (2008) R: A language and environment for statistical computing. R Foundation for Statistical Computing, Vienna, Austria. ISBN 3-900051-07-0, URL http://www.R-project.org.

[45] Davidson EA, Stark JM, Firestone MK (1990) Microbial production and consumption of nitrate in an annual grassland. Ecology 71: 1968–1975.

[46] Verhagen FJM, Laanbroek HJ, Woldendorp JW (1995) Competition for ammonium between plant roots and nitrifying and heterotrophic bacteria and the effects of protozoan grazing. Plant and Soil 170: 241–250.

[47] Cabrera ML, Kissel DE, Vigil MF (2005) Nitrogen mineralization from organic residues: research opportunities. Journal of Environmental Quality 34: 75–79. 15647536 10.2134/jeq2005.0075

[48] Perakis SS, Hedin LO (2001) Fluxes and fates of nitrogen in soil of an unpolluted old-growth temperate forest, southern Chile. Ecology 82: 2245–2260.

[49] Blagodatskaya EV, Blagodatsky SA, Anderson TH, Kuzyakov Y (2009) Contrasting effects of glucose, living roots and maize straw on microbial growth kinetics and substrate availability in soil. European Journal of Soil Science 60: 186–197.

[50] Phillips RP, Finzi AC, Bernhardt ES (2010) Enhanced root exudation induces microbial feedbacks to N cycling in a pine forest under long-term CO2 fumigation. Ecology Letters 14: 187–194. 21176050 10.1111/j.1461-0248.2010.01570.x

[51] Herman DJ, Jaeger KK, Schwartz CH, Firestone E (2006) Root influence on nitrogen mineralization and nitrification in rhizosphere soil. Soil Science Society of America Journal 70: 1504.

[52] Frank DA, Groffman PM (2009) Plant rhizospheric N processes: what we don't know and why we should care. Ecology 90: 1512–1519. 19569366 10.1890/08-0789.1

